# Authentic leadership, perceived insider status, error management climate, and employee resilience: A cross-level study

**DOI:** 10.3389/fpsyg.2022.938878

**Published:** 2022-09-09

**Authors:** Xu Li, Jianyu Zhang

**Affiliations:** Business School, Tianjin University of Finance and Economics, Tianjin, China

**Keywords:** employee resilience, authentic leadership, perceived insider status, error management climate, cross-level effect

## Abstract

Employee resilience is of great significance for organizations to resist pressures, overcome crises, and achieve sustainable development. However, existing research has largely failed to explore its situational triggers. Drawing on social information processing theory and social exchange theory, a cross-level study was conducted to theorize the underlying mechanisms through which authentic leadership facilitates employee resilience. Based on a two-wave time-lagged design, the data were obtained from 85 team leaders and 417 employees in China. The results of the cross-level model showed that authentic leadership was positively related to employee resilience. Perceived insider status and error management climate both played a partial mediating role in linking authentic leadership and employee resilience. Error management climate positively moderated the relationship between perceived insider status and employee resilience. This paper not only contributes to revealing the cross-level effect of authentic leadership on employee resilience but also provides some managerial practices.

## Introduction

With the turbulent business environment and fierce competition, as well as the occurrence of accidents such as natural disasters and industrial accidents, especially the cruel Covid-19 pandemic, organizations need to survive risks and crises. However, the survival and sustainable development of organizations depend on the ability of the organization and its employees to withstand and adapt to major challenges or inevitable adversity, that is, on their resilience (Lengnick-Hall et al., 2011; Näswall et al., 2019). Moreover, employees are the cornerstone of organizational development (Chen et al., 2008), and the willpower and behaviors of employees in crises are of great significance for organizations to resist pressure and promote performance and sustainability (King et al., 2016). In this context, employee resilience has attracted much attention from academia (Bardoel et al., 2014; Näswall et al., 2019). Employee resilience is one kind of developable capacity in working life (Kuntz et al., 2017), which can be supported and facilitated by organizations, to rebound or bounce back from adversity, conflict, and failure in response to dynamic and challenging environments (Zhu et al., 2019). Previous research has shown that employee resilience relates to positive outcomes of individuals and organizations, such as higher job performance (Cooper et al., 2019), job satisfaction (Youssef and Luthans, 2007), job wellbeing (Harms et al., 2018; Bhattacharyya et al., 2019; Iqbal and Piwowar-Sulej, 2021), innovativeness (Sweetman et al., 2011), employee engagement (Cooke et al., 2016), organizational commitment (Shin et al., 2012), and lower turnover intention (Shin et al., 2012). Employee resilience also has been acknowledged as a vital competitive advantage beyond social and economic resources in organizations (Rego et al., 2019; Zhu et al., 2019). Therefore, given the importance of employee resilience, how to activate and enhance it has become a very valuable and important issue.

Scholars have found that employee resilience correlates with positive traits of employees (Sun et al., 2011; Kuntz et al., 2017) and work environment (Caniëls and Baaten, 2019). Among them, leadership has been identified as a critical contextual factor affecting employees’ responses in the workplace (Zhu et al., 2019). Previous studies have explored the influence of humble leadership (Zhu et al., 2019), paradoxical leadership (Franken et al., 2020), and transformational leadership (Djourova et al., 2020) on employee resilience. Different from humble leaders who emphasize viewing themselves and their subordinates objectively (Owens and Hekman, 2012), paradoxical leaders who concentrate on balancing multiple, and often contradictory objectives (Zhang et al., 2015), and transformational leaders who focus on intellectual stimulation and inspirational motivation (Parveen and Adeinat, 2019), the authentic leaders focus on the combination of positive psychological abilities and a highly developed organizational situation to foster positive behaviors in leaders and subordinates (Avolio et al., 2004; Avolio and Gardner, 2005). As a positive and open leadership style, authentic leadership has positive psychological capacities of confidence, hope, and optimism (Avolio and Gardner, 2005). It may provide more effective support and help to employees through establishing transparent, trusting, and collaborative relationships, which are particularly important for employees to resist pressure and deal with setbacks in tough times (Todt et al., 2019). These also increase the likelihood for employees to facilitate greater resilience (Teo et al., 2017). However, there has been limited analysis on how authentic leadership affects employee resilience, leaving a research gap that merits closer examination.

Prior studies on leadership and employee resilience were based on a single individual level (Zhu et al., 2019; Franken et al., 2020), which may hinder the accumulation of knowledge about the cross-level influence of team leadership on employee resilience and is not conducive to our systematic understanding on the impact of the group-level phenomenon on employee resilience. Indeed, employees are nested within a particular work group or team (Zhang, 2010), and employee resilience is the result of the interplay of individual and situational factors (Näswall et al., 2019). Meanwhile, previous studies proposed that leaders could exert significant influence on employees’ behaviors and responses by influencing employees’ cognition and other factors at individual level, as well as by a certain group atmosphere at the group level (Walumbwa et al., 2010; Zhou and Pan, 2015). Therefore, our research proposes a multilevel approach to examine the cross-level effect of authentic leadership on employee resilience by considering employees as groups of followers in relation to team leaders.

One of the factors from the individual level is perceived insider status (PIS) reflecting the psychological cognitive state of employees as insiders within a particular organization (Stamper and Masterson, 2002). Empirical evidence has found that PIS is positively associated with employee resilience based on the social exchange theory (Blau, 1964), considering that higher PIS stimulates employees to contribute more efforts, complete tasks beyond their roles, and solve problems confronted at work (Zhu et al., 2019). Prior research also pointed out that authentic leadership could have a positive impact on employees’ affective attachment to and cognitive state with their organizations (Avolio et al., 2004; Leroy et al., 2012). Therefore, we propose that PIS will be the mediating variable that links authentic leadership to employee resilience.

Among other predictive factors from the team level, team error management climate (EMC) reflects the sharing perception of employees relating to communicating errors, sharing error knowledge and learning from errors, and helping in faulty situations (van Dyck et al., 2005). It has been proven that EMC could motivate employees to deal with difficulties or challenges creatively (Chen et al., 2020), which may lead to resilient reactions in the workplace (Caniëls and Baaten, 2019). Gardner et al. (2011) pointed out that authentic leaders could acknowledge guilt and errors, accept responsibility for their actions and mistakes, and avoid blaming others for errors. This contributes to a learning-from-errors orientation in a team context, which could be captured by EMC (Farnese et al., 2019). Based on social information processing theory, leaders, as vital sources of social information, are helpful to shape employees’ perceptions about the work environment, and, in turn, have an impact on employees’ responses (Salancik and Pfeffer, 1978). Previous research also indicated that team leadership may influence employee outcomes through team climate (Walumbwa et al., 2008, 2010). Thus, we examine the mediating role of EMC in authentic leadership and employee resilience linkage. Furthermore, Parker et al. (2010) pointed out that team climate could play a role of boundary condition in the process of individuals engaging in active behaviors. Following this idea, we propose that EMC may also be a possible moderator to explain the effect of perceived insider status on employee resilience.

In summary, as employee resilience reflects a capacity that can be developed, this study is designed to examine the relationship between team-level authentic leadership and employee resilience, and identify the roles of perceived insider identity and EMC in this relationship. This study provides important implications for team managerial practices.

## Theory and hypotheses

### Social information processing theory

Social information processing theory (Salancik and Pfeffer, 1978) suggests that individuals’ perceptions, attitudes, and behaviors are often influenced by complex and ambiguous social environmental information, which provides cues that individuals may use to construct and interpret event. In the workplace, leaders are viewed as vital sources of social information for employees, because they are in high status and have consistent interactions with subordinates they lead (Yaffe and Kark, 2011). Employees could regulate their attitudes and behaviors by perceiving and interpreting cues or signals released by leaders (Salancik and Pfeffer, 1978). Thus, leader behaviors will deeply influence employees’ subsequent perceptions, attitudes, and behaviors. Prior studies on social information processing theory have also validated its implications on organizational leadership studies (Ou et al., 2014; Lu et al., 2018; Zhong et al., 2019; Zhang and Song, 2020).

From the perspective of social information processing theory, we argue that the statements and behaviors of authentic leadership in response to environmental challenges or changes provide a cue to subordinates and play a vital role in affecting employee resilience. Social information processing theory explains that social interactions with significant others, such as leaders and peers, determine cognitive and behavioral responses (Ou et al., 2014; Boekhorst, 2015; Chen et al., 2020). Further, we argue that increased positive exchanges of information from authentic leaders may shape employees’ perceptions about their social status in the organization and work environment climate, which, in turn, influence employee resilience.

### Authentic leadership and employee resilience

“Authenticity” as a construct dates back to the ancient Greeks philosophy of “be true to oneself” (Avolio et al., 2004), which reflects the congruence of behaviors and true self. With the development of positive psychology and positive organizational behavior, authentic leadership is regarded as the root construct of positive leadership (Avolio and Gardner, 2005), and it is often associated with an inclusive, positive, ethical, and supportive organizational context (Gardner et al., 2005). Authentic leaders are those who are confident, hopeful, optimistic, resilient, and of high moral character (Avolio and Gardner, 2005). They have a strong sense of self-worth and belief to foster greater self-regulated positive behaviors and contribute to positive self-development in themselves and their followers (Avolio et al., 2004). Authentic leadership is generally categorized into four related dimensions: self-awareness, balanced processing, relational transparency, and internalized moral perspectives (Walumbwa et al., 2008). Self-awareness refers to leaders’ awareness of their strengths and weakness, as well as their impact on subordinates (Avolio and Gardner, 2005). Balanced processing involves being unbiased in considering all relevant information before coming up with a fair decision (Gardner et al., 2005). Relational transparency refers to that leaders present their true selves to their followers, which helps foster an open and transparent atmosphere, and build trusting relationships with their subordinates (Gardner et al., 2005). Internalized moral perspective is related to leaders’ behaviors that are compatible with their moral values and beliefs, and will not violate their own moral standards due to external pressure (Walumbwa et al., 2008).

Previous studies have demonstrated that authentic leadership has a positive impact on employees’ responses, such as job performance (Peterson et al., 2012), creativity (Rego et al., 2012), and work engagement (Bamford et al., 2013), etc. Authentic leadership is also proclaimed the catalyst to organizational performance, especially in an uncertain business environment, and heralded as an answer to a broad range of ethical and environmental challenges (Alvesson and Sveningsson, 2013). According to prior studies, it is deduced that authentic leadership has a positive impact on employee resilience. In specific, first, authentic leaders are hopeful, positive, resilient, and optimistic (Avolio and Gardner, 2005). In the face of changes or challenges, they not only withstand but also thrive (Hannah et al., 2011). In accordance with social information processing theory, authentic leaders as important social cues send positive social information about coping with changes or challenges to employees. With the role modeling of authentic leaders, subordinates will imitate their leaders when it comes to demonstrating their positive emotion and perception about challenges, increasing their adaption to changes, as well as achieving growth from failures (Bandura and Walters, 1977). Second, authentic leaders foster transparency in engaging with subordinates, and accept different opinions and views, which provide psychological assistance, enable subordinates to feel a full sense of trust and safety, and promote employees to learn and convey unconventional thoughts freely (Schuckert et al., 2018). These can be viewed as important antecedents for employee resilience (Zhu et al., 2019). Finally, authentic leadership can organize high standards of moral. It has also been assumed that the moral responsibilities of leaders are to generate employee resilience, especially in the time of crisis (Välikangas, 2020). Therefore, the following hypothesis was proposed:

*H1:* Authentic leadership is positively related to employee resilience.

### Mediating role of perceived insider status

Perceived insider status (PIS) refers to the extent to which employees perceive themselves as insiders within a particular organization (Stamper and Masterson, 2002), reflecting employees’ cognition of their social status in the organization and emphasizing employees’ sense of belonging to the organization (Wang et al., 2017). According to social information processing theory, PIS could be affected by social information cues from leader behaviors. Based on a relationship model of authority in groups, Tyler and Lind (1992) also proposed that treatment by the supervisor influences one’s perception of social standing in the work group. Previous studies pointed out that high-quality work relationships have an important impact on PIS (Schaubroeck et al., 2017). Authentic leaders care about and respect subordinates, and foster high-quality leader-subordinate relationships by exhibiting openness to different points, establishing transparent and trusting relationships with employees, and focusing on the growth of employees(Gardner et al., 2005; Sengupta et al., 2021). These positive characteristics and behaviors send signals and cues to employees that they are accepted by the organization, enhancing their sense of belonging to the organization (Rhoades and Eisenberger, 2002). Further, these can facilitate the shaping of employees’ perceptions as insiders within the organization. Moreover, employees in tough times often crave more support and attention from their leaders (Näswall et al., 2019). Authentic leaders correlate with higher levels of information sharing with employees, guide employees to actively face difficulties, and sincerely provide help and support to employees (Todt et al., 2019). As such, employees are more likely to feel supported and easily perceive themselves as insiders in an organization (Lapalme et al., 2009).

Employees with a higher sense of insider status have a stronger affective and behavioral attachment to the organization (Wang et al., 2017). They also have a stronger sense of belonging to the organization and being accepted, trusted, and supported by the organization (Stamper and Masterson, 2002). Previous research has already proposed the positive impact of PIS on employee resilience (Zhu et al., 2019). In specific, according to social exchange theory, to reciprocate the organizations’ accepting, trusting, and supporting, employees deem that it is their role responsibilities to promote the long-term development of the organization, and feel obligated to contribute efforts beyond that required by the jobs with the expectation (Stamper and Masterson, 2002). Especially in a crisis situation, higher PIS correlates with better task performance (Wang and Kim, 2013), solving problems positively, adapting to challenges, and further, engaging in resilient reactions (Zhu et al., 2019). Thus, we deem that authentic leadership has a positive effect on employee resilience through perceived insider status and propose the following:

*H2:* Perceived insider status mediates the relationship between authentic leadership and employee resilience.

### Mediating role of error management climate

Every organization is confronted with errors. A growing body of research suggests that an organizational climate, whose continuous improvement mechanisms include the open discussion of problems by employees and learning from errors, may be positively associated with work engagement and resilience in employees (Huang and Luthans, 2015; Meneghel et al., 2016). Error management climate (EMC) reflects organizational practices and procedures relating to communicating errors, sharing error knowledge, learning from errors, helping in error situations, and quickly detecting, analyzing, and resolving errors (van Dyck et al., 2005). In this study, we focus on EMC at the team level, and deem that EMC may play a mediating role between authentic leadership and employee resilience at the team level.

As social information processing theory posits (Salancik and Pfeffer, 1978; Gu et al., 2016; Chen et al., 2020), employees rely on cues or signals from leaders to confirm how they understand the environment or climate in the organization and then regulate their attitude and behaviors accordingly to suit the environment. Previous studies also demonstrate that leaders, as “climate engineers,” play an important role in the development of team climate (Naumann and Bennett, 2000). Meanwhile, leadership style and perceptions of leaders about errors affect the formation of EMC (Cigularov et al., 2010). Authentic leadership has a positive attitude toward errors, regards errors as a source of learning and growth, acknowledges personal faults, and encourages employees to detect errors in time and take responsibility for their own behaviors (Farnese et al., 2019). These may create a constructive and supportive working atmosphere and provide signal guidance to employees that organizational approaches to errors are positive, and making mistakes is not unusual, while learning and growing from mistakes is more important than blaming (Owens and Hekman, 2012). In addition, authentic leaders are self-aware, transparent and open in their communication with employees. Employees are willing to exchange information and knowledge about errors with others, so as to learn from teammates’ experience and errors (Gardner et al., 2011). All of these contribute to a learning-from-errors orientation in the team and enhance EMC.

Furthermore, we speculate that EMC is positively related to employee resilience. A team with high-level EMC regards errors as the common phenomenon and learning opportunities, allows employees to make mistakes, and encourages them to reflect, learn and discuss about errors (van Dyck et al., 2005). This not only prevents other teammates from making similar mistakes, but also creates a positive and safe team climate in which followers feel comfortable and do not worry about being punished and mocked at for their mistakes (Edmondson and Lei, 2014). Further, this reduces employees’ work pressure, psychological burden, and emotional exhaustion, and makes them believe that they are capable of coping with the difficulties and challenges at work (van Dyck et al., 2005). They also would like explore more ways to solve the problems (Frese and Keith, 2015). In contrast, employees are likely to be anxious about being punished for their mistakes in the team with low-level EMC. When facing adversities, they tend to get stuck in a rut and lack the courage to try new ways to avoid errors, which may lead to work stress and fatigue (Farnese et al., 2019) and ultimately impact employee resilience negatively. As such, we propose:

*H3:* EMC mediates the relationship between authentic leadership and employee resilience.

### Moderating role of error management climate

Higher perceived insider status is an important reason for enhancing employee resilience, however, employees are nested in a specific team, and employee resilience will be affected by team atmosphere. Huang and Luthans (2015) also pointed out that, compared with focusing on a single level of influence, arguing the interaction between an individual and the environment can more comprehensively reveal individual attitudes and behavioral characteristics. Pressure, frustrations, and difficulties at work have a negative impact on employees. However, positive team climate can provide a context in which individuals feel safe (Cigularov et al., 2010), and are willing to overcome difficulties and grow from adversities (Zhang and Song, 2020). Thus, we consider EMC as the boundary condition of the relationship between perceived insider status and employee resilience.

Error management climate creates a supportive working environment for learning from errors, discussing errors openly, and sharing knowledge (Maurer et al., 2017). Instead of punishing employees who make mistakes, teams with high-level EMC try to understand their errors, help employees deal with errors, and learn from errors (Guchait et al., 2018). As a result, team members do not fear being blamed for making mistakes. Further, these team members would feel that they should contribute more and exhibit more positive behaviors, such as active learning, expanding resources, and problem solving (van Dyck et al., 2005; Frese and Keith, 2015). These are consistent with the positive psychology and behaviors of employees with a higher sense of perceived insider status in tough times. Perceived insider status reveals the positive psychological cognitive factors that enhance employee resilience from individual-level factors. However, employee resilience is the result of the interaction between individual-level factors and team-level factors (Näswall et al., 2019). Thus, EMC can provide a context that strengthens the influence of perceived insider status, which, in turn, enhances employee resilience. On the contrary, if a team has a low-level EMC, emphasizes punishment, and blames for errors, team members tend to view errors negatively (van Dyck et al., 2005). In such an environment, even if employees consider themselves as insiders of the organization, they will still be under high pressure, feel nervous and anxious about making mistakes, and are unwilling to face difficulties and challenges at work, resulting in low employee resilience. Accordingly, we propose:

*H4:* EMC positively moderates the relationship between perceived insider status and employee resilience, i.e., the higher the level of EMC, the stronger the positive relationship between perceived insider status and employee resilience.

### Conceptual model

**Figure 1:**
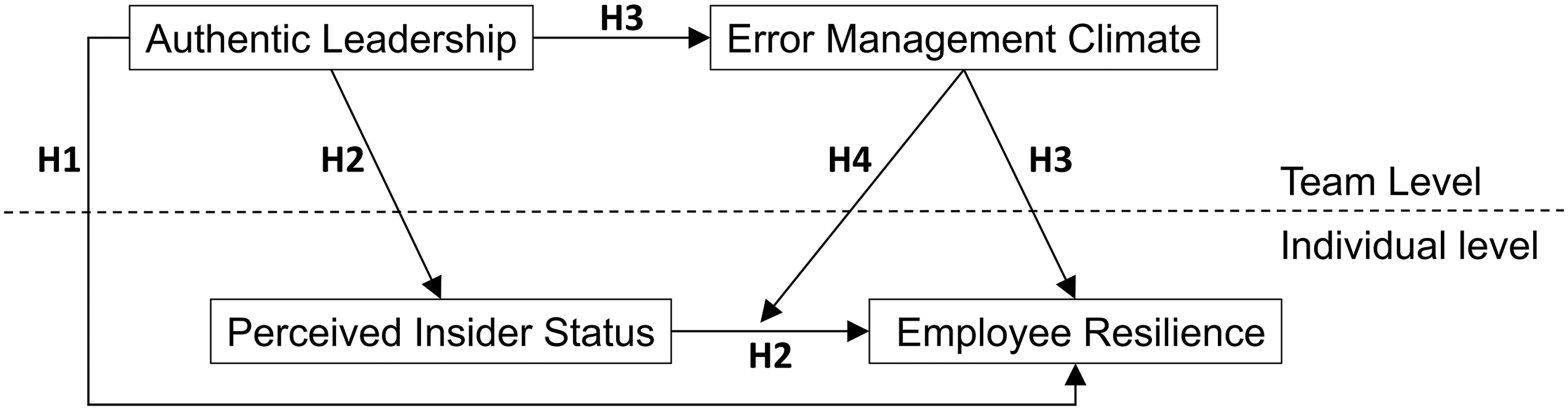
Theoretical model.

The theoretical model is presented in Figure 1.

## Materials and methods

### Sample and data collection

We collected the data from companies located in Beijing, Jiangsu, Zhejiang, Shanghai, and Guangzhou of China, mainly involving in the service industry, manufacturing industry, information technology industry, and pharmaceutical research and development industry. Within the chosen organizations, our survey further focused on selecting leaders and subordinates from established teams. We defined a team to be composed of one leader and at least five members who reported directly to the leader.

Before the investigation, we contacted the company manager and human resource department to introduce the purpose and procedure of the research in detail, and stated that the survey was only for academic research and did not involve commercial confidential information. Then, with the cooperation of human resource department, the questionnaire was immediately distributed to respondents, all of whom were voluntary. To increase honest responses, we provided each participant with a randomly generated code as their identification number when they filled out their questionnaires, which allowed us to match subordinates’ surveys with their team leaders while remaining confidential.

We administered the survey at two time points. At the first time point, subordinates were asked to assess authentic leadership, perceived insider status, error management climate, and demographic variables. At the second time point (4 weeks later), team leaders were asked to assess employee resilience and team information. The questionnaires were sent to 90 team leaders and 450 employees, with a leader-to-subordinate ratio of 1:5, and data were collected by leader-subordinate pairing. After excluding invalid questionnaires such as incomplete filling, obvious logical errors, and unmatched ones, 85 leader questionnaires (response rate 94.4%) and 417 employee questionnaires (response rate 92.7%) were received. The average leader-to-subordinate ratio was 1:4.9.

Among the 417 subordinates, 63.5% were female, and 36.5% were male. Most of the respondents (65.5%) were under 30 years of age, 32.4% were aged between 31 and 40. In terms of education level, 65.2% had a bachelor degree or above. Among the 85 leaders, 45.9% were female, and 54.1% were male. In terms of their age, 52.9% were aged between 31 and 40, and 25.9% were aged between 41 and 50. In terms of education level, 83.5% had a bachelor degree or above. Team size was concentrated in 6–10 people and 11–20 people, accounting for 31.8% and 34.1%, respectively.

### Measures

All the scales used in the current study were derived from mature scales in relevant literature. To ensure the validity of the scales, we translated the English scale to Chinese following standard back-translation procedures (Brislin, 2016). A Likert five-point scale was used for all the measures, with 1 indicating strongly disagree and 5 indicating strongly agree.

### Authentic leadership

We used a 16-item scale developed by Walumbwa et al. (2008) to measure authentic leadership. The scale encompassed four dimensions: self-awareness, balanced processing, relational transparency, and internalized moral perspectives. Two sample items are “My team leader openly shares information with others” and “My team leader seeks feedback to improve interactions with others.” Cronbach’s α was 0.914 in this study.

### Perceived insider status

We used a six-item scale developed by Stamper and Masterson (2002) to measure perceived insider status. A sample item is “I feel like I’m a part of the team.” Cronbach’s α was 0.886 in this study.

### Error management climate

We used a 16-item scale developed by Cigularov et al. (2010) to measure team-level EMC, which encompassed four dimensions: learning from errors, error communication, thinking about errors, and error competence. Sample items included “Errors are a guide to the team’s subsequent work” and “Team members are willing to learn from others’ errors in order to achieve the goals.” Cronbach’s α was 0.888 in this study.

### Employee resilience

We used a nine-item scale developed by Näswall et al. (2019) to measure employee resilience. A sample item was “I regard challenges at work as opportunities for growth.” Cronbach’s α was 0.920 in this study.

### Control variables

This study chose employees’ gender, age, educational level, leaders’ tenure, and time size as the main control variables according to previous studies (Hirak et al., 2012; Wu and Parker, 2017; Caniëls and Baaten, 2019; Zhang and Song, 2020).

## Results

### Data aggregation test

Authentic leadership and EMC were team-level constructs, but the scales were completed by employees. Hence, we used internal consistency (Rwg), within-group reliability (ICC1), and reliability of group mean (ICC2) to test whether aggregating individual members’ rating to the team level was appropriate. The Rwg, ICC1, and ICC2 of authentic leadership were 0.944, 0.494, and 0.830, respectively. The Rwg, ICC1, and ICC2 of EMC were 0.964, 0.577, and 0.872, respectively. These three indicators (Rwg, ICC1, and ICC2) of authentic leadership and EMC exceeded the acceptable values of 0.70, 0.12, and 0.70, supporting aggregation.

### Confirmatory factor analysis

We conducted a confirmatory factor analysis (CFA) by using Mplus 8.3 to check the discriminant validity among the variables (authentic leadership, perceived insider status, error management climate, and employee resilience). Given that authentic leadership and EMC each contained four dimensions, and involved a large number of items, items in each sub-dimension were parceled to enhance the reliability and normality of the resulting measure prior to performing CFA (Little et al., 2002). As shown in Table 1, the hypothesized four-factor model was a better fit into the data than other alternative models (*χ*^2^/*df* = 1.682, RMSEA = 0.040, SRMR = 0.038, CFI = 0.967, TLI = 0.963), which indicates that variable discriminant validity is verified. In this study, employee resilience was evaluated by the team leader at the time point 2, while all other variables were self-reported by employees, which may lead to common method variance (CMV). Therefore, we used the unmeasured latent method factor approach (Podsakoff et al., 2003) and added one common factor to construct the five-factor model based on the four-factor model (Table 1). Compared with the four-factor model, the five-factor model fitted better, but the fit indices did not improve significantly, which indicated that CMV was not a serious problem in this study.

**Table 1:** Confirmatory factor analysis.

Model	*χ*2	*df*	*χ*2/*df*	RMSEA	SRMR	CFI	TLI
Five factors: AL, PIS, ERC, ER, CMV	317.613	202	1.572	0.037	0.034	0.975	0.969
Four factors: AL, PIS, EMC, ER	376.673	224	1.682	0.040	0.038	0.967	0.963
Three factors: AL + PIS, EMC, ER	952.471	227	4.196	0.088	0.085	0.845	0.827
Two factors: AL + PIS + EMC, ER	1309.976	229	5.720	0.106	0.102	0.769	0.745
Single factor: AL + PIS + EMC + ER	2115.643	230	9.198	0.140	0.122	0.597	0.557

Individual level *n* = 417, team level *n* = 85; AL, authentic leadership; PIS, perceived insider status; EMC, error management climate; ER, employee resilience; CMV, common factor; +, represents the combination of factors.

### Descriptive statistics

Table 2 shows the main characteristics of the samples, including means, standard deviations, and variable correlations at both individual and team levels. Perceived insider status was significantly related to employee resilience (*r* = 0.439, *p* < 0.01), and authentic leadership was significantly related to EMC (*r* = 0.267, *p* < 0.01). These results preliminarily verified the relevant hypotheses of this study and laid a foundation for further analysis.

**Table 2:** Means, standard deviations, and correlations among variables.

Variable	*M*	SD	1	2	3	4
*Individual-level*						
Gender	1.640	0.482				
Age	1.370	0.525	−0.040			
Education	1.690	0.543	0.077	0.177^**^		
PIS	4.282	0.538	0.031	0.060	0.077	
ER	4.121	0.548	0.019	0.071	0.126^**^	0.439^**^
*Team-level*						
Leader Tenure	2.280	0.779				
Team Size	2.590	0.957	0.241^**^			
AL	3.765	0.598	0.187^**^	0.063		
EMC	4.045	0.466	0.127^**^	0.133^**^	0.267^**^	

Individual level *n* = 417, team level *n* = 85; AL, authentic leadership; PIS, perceived insider status; EMC, error management climate; ER, employee resilience.

** *p* < 0.01.

### Hypothesis testing

The variables in this study involved two levels (team-level and individual-level), hence, a cross-level model was used to test the hypothesis. First, we constructed a null model to analyze the within-group variance and between-group variance of employee resilience. The results showed that the within-group variance (σ2) was 0.176 and the between-group variance (τ00) was 0.127. The ICC1 was 0.419, higher than the acceptable value of 0.059, indicating that 41.9% of the variance in employee resilience was at team level. Therefore, the data in this study was suitable for cross-level analysis.

Then, we used Mplus 8.3 for cross-level analysis and the results are shown in Table 3. As shown in Model 4, authentic leadership was positively related to employee resilience (*γ*_01_ = 0.407, *p* < 0.01) after the addition of control variables (employee gender, age, educational level, leadership tenure, and time size) and authentic leadership, thus, hypothesis 1 (the main effect) was verified. From model 1, we can see that authentic leadership was positively related to perceived insider status (*γ*_01_ = 0.423, *p* < 0.01). From model 2, we can see that authentic leadership was positively related to EMC (γ_01_ = 0.334, *p* < 0.01). In model 5, after authentic leadership, perceived insider status and EMC were added to explain employee resilience, the influence coefficient of authentic leadership on employee resilience changed from *γ*_01_ = 0.407 (*p* < 0.01) to *γ*_01_ = 0.163 (*p* < 0.05). This indicated that perceived insider status played a partially mediating role between the authentic leadership and employee resilience (*γ*_10_ = 0.298, *p* < 0.05). Thus, hypothesis 2 was confirmed. Meanwhile, EMC played a partially mediating role between authentic leadership and employee resilience (*γ*_02_ = 0.355, *p* < 0.01), and hypothesis 3 was also confirmed.

**Table 3:** The results of main effect and mediating effect.

Variable	PIS	EMC	ER		
	Model 1	Model 2	Model 3	Model 4	Model 5
Intercept	2.297^**^	2.637^**^	3.449^**^	2.078^**^	0.473
*Individual-level*					
Gender	0.011	0.025	0.017	0.014	0.010
Age	0.040	0.033	0.031	0.035	0.016
Education	0.069	0.010	0.105^*^	0.095^*^	0.070
PIS					0.298^*^
*Team-level*					
Leader Tenure	0.072	0.015	0.132^*^	0.075	0.048
Team Size	0.015	0.046	0.047	0.041	0.020
AL	0.423^**^	0.334^**^		0.407^**^	0.163^*^
EMC					0.355^**^
Variance decomposition					
Within-group variance(σ2)	0.131^**^	0.091^**^	0.173^**^	0.173^**^	0.162^**^
Between-group variance(τ00)	0.109^**^	0.115^**^	0.110^**^	0.077^**^	0.047^**^

Individual level n = 417, team level n = 85; AL, authentic leadership; PIS, perceived insider status; EMC, error management climate; ER, employee resilience.

* *p* < 0.05 and

** *p* < 0.01.

**Figure 2:**
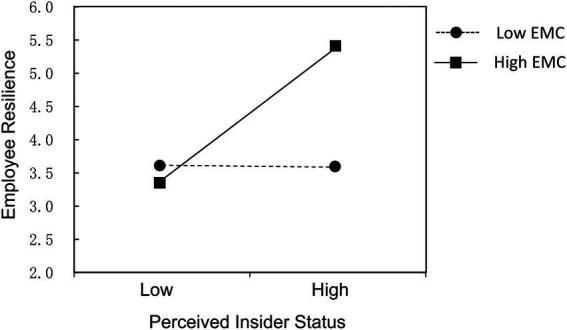
Moderating role of EMC on the relationship between perceived insider status and employee resilience.

To further verify the mediating effect of perceived insider status and EMC, we used Monte Carlo simulation for robust test. The results showed that the indirect effect of authentic leadership on employee resilience through perceived insider status was 0.126 (95% CI 0.007–0.261), and the confidence intervals did not include 0, which meant the mediating effect of perceived insider status was significant, and hypothesis 2 was further verified. Likewise, the indirect effect of authentic leadership on employee resilience through EMC was 0.117 (95% CI 0.023–0.249), and the confidence intervals did not include 0, which meant the mediating effect of EMC was significant, and hypothesis 3 was further verified. Table 4 shows the moderating effects of EMC between perceived insider status and employee resilience. From model 2, we can see that perceived insider status was positively related to employee resilience (*γ*_10_ = 0.500, *p* < 0.01). In model 3, perceived insider status and EMC were added to explain employee resilience. From model 4, we can see that the interaction term of perceived insider status and EMC was significantly and positively related to employee resilience (*γ*_11_ = 0.510, *p* < 0.05), indicating that EMC positively moderated the relationship between perceived insider status and employee resilience. Figure 2 further illustrates the moderating effect of EMC between perceived insider status and employee resilience. The relationship between perceived insider status and employee resilience is stronger when EMC is high than when it is low. Therefore, hypothesis 4 was further verified.

**Table 4:** The moderating effect analysis of EMC.

Variable	ER			
	Model 1	Model 2	Model 3	Model 4
Intercept	3.449^**^	3.683^**^	0.580	3.766^**^
*Individual-level*				
Gender	0.017	0.009	0.011	0.009
Age	0.031	0.020	0.013	0.010
Education	0.105^*^	0.085^*^	0.075	0.069
PIS		0.500^**^	0.368^**^	0.377^**^
*Team-level*				
Leader Tenure	0.132^*^	0.066	0.058	0.026
Team Size	0.047	0.038	0.019	0.044
EMC			0.400^**^	0.368^**^
PIS × EMC				0.510^*^
Within-group variance(σ2)	0.173^**^	0.161^**^	0.162^**^	0.162^**^
Between-group variance(τ00)	0.110^**^	0.070^**^	0.051^**^	0.046^**^

Individual level n = 417, team level n = 85; AL, authentic leadership; PIS, perceived insider status; EMC, error management climate; ER, employee resilience.

* *p* < 0.05 and

** *p* < 0.01.

## Discussion

Employee resilience is crucial for promoting the development of organizations, especially for coping with crises and challenges in an uncertain environment (Fisher et al., 2016; King et al., 2016). There is growing interest in understanding the nature and antecedents of employee resilience (Barasa et al., 2018; Isidro and Calleja, 2021). Leaders play a critical role in employees’ responses to challenging and stressful environments (Harland et al., 2005; Teo et al., 2017; Salehzadeh, 2019). Hence, based on social information processing theory and social exchange theory, our study explores how and when team-level authentic leaders drive followers’ resilience drawing upon a sample of 85 leaders and 417 employees in China through two waves of data collection. The results of the empirical study support all the proposed hypotheses and the main findings are as follows:

First, authentic leadership is positively correlated with employee resilience. Employees who perceive their leaders’ authenticity and sincerity are more likely to feel trusted (Gardner et al., 2005) and emotionally supported (Peterson et al., 2012), which motivate them to exhibit more resilience by engaging in and handling encountered challenges. These findings also agree with previous research holding that authentic leaders are well positioned to impact levels of positivity and performance in followers when performing in more stressful contexts (Avolio et al., 2004; Ilies et al., 2005; Peterson et al., 2012).

Second, perceived insider status and EMC partially mediate the relationship between authentic leadership and employee resilience. Team-level authentic leaders could influence employee resilience through individual- and team-level mechanisms simultaneously. By accepting responsibility for their own actions, showing openness to new ideas, and establishing transparent and trusting relationships with subordinates (Gardner et al., 2011; Nielsen et al., 2013), authentic leadership facilitates the shaping of employees’ perceived insider status, as well as team EMC in the workplace, which encourages employees to further develop resilience.

Third, EMC positively moderates the relationship between perceived insider status and employee resilience. Compared with low-level EMC, the positive relationship between perceived insider status and employee resilience is stronger under high-level EMC, which highlights the positive approaches to errors and creates an inclusive and supportive environment (Gronewold et al., 2013; Farnese et al., 2019). Although the mentioned above has demonstrated that EMC act as a mediator between authentic leadership and employee resilience, EMC could also serve as a cross-level moderator of the relationship between perceived insider status and employee resilience. This is in line with considerable previous research about cross-level mediating and moderating effects of group process variables (Walumbwa et al., 2008, 2010; Ehrhart, 2010).

### Theoretical implications

Our study provides several theoretical implications. First, our research explores the key role of team-level authentic leadership in promoting employee resilience. In the workplace, leadership has always been regarded as a key antecedent variable to predict employee’ responses (Williams et al., 2010; Hsiung, 2012). Previous studies have explored the impact of different leaders on employee resilience, such as humble leadership (Zhu et al., 2019), paradoxical leadership (Franken et al., 2020). However, positive leadership, who has positive psychological abilities and could build high-quality leader-member relationship, is also particularly of great significance in tough times (Avolio and Gardner, 2005). Hence, we propose team-level authentic leadership as an antecedent variable of employee resilience and explore the cross-level effects of authentic leadership on employee resilience, which enriches the literature on antecedents of employee resilience and further broadens the knowledge about the leadership-employee relationship.

Second, by verifying the mediating effect of perceived insider status and EMC in the linkage between authentic leadership and employee resilience, this study could capture a more complete picture of how authentic leadership influences employee resilience through individual- and team-level mechanisms. Previous studies on the relationship between leadership and employee resilience have mostly focused on individual-level mechanisms (i.e., positive emotion, perceived organizational support; Harland et al., 2005), few studies have addressed the team-level mechanisms. This study amplifies authentic leadership effectiveness through influencing individual-level perception and contextual mechanism, comprehensively opening the “black box” of mechanism between authentic leadership and employee resilience from a multi-level perspective.

Finally, this study demonstrates the cross-level moderating effect of EMC between perceived insider status and employee resilience. This result is consistent with the cross-level theoretical model of Walumbwa et al. (2010), which examined climates’ cross-level interactions with individual-level attitudes in predicting employee behaviors. Employees are nested within specific teams, and employee resilience is the result of the interplay of individual and situational factors (Näswall et al., 2019). However, few studies have revealed the interactive effects in predicting employee resilience. In this study, we explore the cross-level moderating role of EMC to clarify the boundary conditions of perceived insider status on employee resilience, contributing to further understand employee resilience from the perspective of the interaction of individual-level and team-level factors.

### Practical implications

Our findings also provide several managerial implications. First, our research that shows the impact of authentic leadership on catalyzing employee resilience in the workplace, indicates the importance of selecting, cultivating, and promoting leaders with authentic features and behaviors. In the modern business society, many organizations choose leaders more based on their performance and competency (Cheng et al., 2021), neglecting the psychological abilities of leaders and construction of high-quality leadership-employee relationship. Our study demonstrates that authentic leadership can facilitate employees’ adaption to changes as well as their recovery from failures, which is vital for organizations to achieve sustainable development, especially in a crisis situation (Rego et al., 2019). Hence, organizations can provide some training programs, learning groups, or individual coaching to cultivate leaders with authentic features, encourage leaders to establish a trusting relationship with subordinates, and communicate with them transparently and openly.

Second, the verified perceived insider status mediation between authentic leadership and employee resilience highlights the significance of facilitating employees’ perceptions as insiders of the organization by authentic leaders, as well as the important role perceived insider status played in activating employee resilience. Team leaders should pay more attention to creating conditions where employees feel very much a part of the team. To achieve this, team leaders need to take some actions, i.e., interact positively with employees, pay attention to employees’ internal needs and sense of belonging to the organization, as well as create a working climate of trust, transparent, safety, and support.

Finally, our findings indicate that EMC seems to be an ideal work environment for promoting employee resilience. Team leaders could cultivate a high-level EMC by creating an inclusive climate for errors, and establishing mutual trust, and mutual assistance among members to facilitate communication and knowledge sharing with errors. Also, team leaders might establish platforms or mechanisms to enhance employees’ awareness of their own orientation toward errors and encourage employees to learn from errors.

### Limitations and directions for future research

Despite these contributions, this study still has some potential limitations. First, we used a cross-sectional design to explore the relationship between authentic leadership and employee resilience, which makes it difficult to effectively clarify the dynamic evolutionary relationship between them. And the level of employee resilience may vary depending on the degree of error or adversity, which cannot be measured by cross-sectional design. In future research, an experimental study can be utilized to explore the causal relationship between authentic leadership and employee resilience, as well as the degree of error or adversity. Second, personality traits also play crucial roles in employee’s behaviors, which were not explored in our study. Future research can explore the moderating effects of employee traits, e.g., self-efficacy, vigor, and critical thinking, or use employee traits as control variables in the research framework. Third, the data of this study were retrieved from enterprises in China, and we did not control the type of company or industry, which may also have influence on employee resilience. We recommend future research in other countries and various type of enterprise to further examine the effect of authentic leadership on employee resilience. Finally, this study explores the mechanism of authentic leadership on employee resilience from individual-level and team-level factors, but lacks variables at organizational-level. Future research on employee resilience can be expanded from the perspective of the interaction of individual-organizational factors or team-organizational factors.

## Conclusion

Research on employee resilience has received a lot of attention. Nevertheless, current studies have primarily investigated the impacts of leader behaviors on employee resilience based on a single individual-level factor while neglecting the effects of group-level factors on employee resilience. Extending this stream of research, in this study, we develop a cross-level model in which team-level authentic leadership influences employee resilience through individual-level cognition (i.e., perceived insider status) and team climate (i.e., EMC) simultaneously. Further, we systematically consider the interactive effect of individual-level cognition and team climate on employee resilience, illustrating that EMC moderates the relationship between perceived insider status and employee resilience, that is, higher-level employee resilience is likely to be enhanced when perceived insider status of employees is accompanied by reinforcement of EMC. Our study highlights the importance of leaders in influencing employees’ responses, especially in challenging and stressful situations, by demonstrating the cross-level effect of authentic leadership on employee resilience. We hope this research sparks further interest in advancing the literature on employee resilience and authentic leadership.

## Data availability statement

The raw data supporting the conclusions of this article will be made available by the authors, without undue reservation.

## Author contributions

XL designed the study, performed the analysis, and wrote the manuscript. JZ collected the data and revised the manuscript. All authors contributed to the article and approved the submitted version.

## Funding

This work was funded by the Project of Humanities and Social Sciences of Ministry of Education in China (18YJA630138).

## Conflict of interest

The authors declare that the research was conducted in the absence of any commercial or financial relationships that could be construed as a potential conflict of interest.

## Publisher’s note

All claims expressed in this article are solely those of the authors and do not necessarily represent those of their affiliated organizations, or those of the publisher, the editors and the reviewers. Any product that may be evaluated in this article, or claim that may be made by its manufacturer, is not guaranteed or endorsed by the publisher.
